# Strategies and Mechanism in Reversing Intestinal Drug Efflux in Oral Drug Delivery

**DOI:** 10.3390/pharmaceutics14061131

**Published:** 2022-05-26

**Authors:** Rong Lu, Yun Zhou, Jinqian Ma, Yuchen Wang, Xiaoqing Miao

**Affiliations:** 1 Marine College, Shandong University, Weihai 264209, China; lurong@sdu.edu.cn (R.L.); zy180618zy@163.com (Y.Z.); 17854160138@163.com (J.M.); w17660520116@163.com (Y.W.); 2 School of Pharmaceutical Sciences, Shandong University, Jinan 250012, China

**Keywords:** efflux transporters, oral drug absorption, functional excipients, inhibitors, inhibiting intestinal drug efflux, nano-preparations

## Abstract

Efflux transporters distributed at the apical side of human intestinal epithelial cells actively transport drugs from the enterocytes to the intestinal lumen, which could lead to extremely poor absorption of drugs by oral administration. Typical intestinal efflux transporters involved in oral drug absorption process mainly include P-glycoprotein (P-gp), multidrug resistance proteins (MRPs) and breast cancer resistance protein (BCRP). Drug efflux is one of the most important factors resulting in poor absorption of oral drugs. Caco-2 monolayer and everted gut sac are sued to accurately measure drug efflux in vitro. To reverse intestinal drug efflux and improve absorption of oral drugs, a great deal of functional amphiphilic excipients and inhibitors with the function of suppressing efflux transporters activity are generalized in this review. In addition, different strategies of reducing intestinal drugs efflux such as silencing transporters and the application of excipients and inhibitors are introduced. Ultimately, various nano-formulations of improving oral drug absorption by inhibiting intestinal drug efflux are discussed. In conclusion, this review has significant reference for overcoming intestinal drug efflux and improving oral drug absorption.

## 1. Introduction

Oral administration is the most practical and popular approach due to its good patient compliance, convenience and low cost compared to other administration routes, and it could accomplish the purpose of local and systemic treatment. However, the oral absorption of some drugs is limited due to poor solubility [1,2], poor intestinal permeability [3], liver first-pass effect and particularly intestinal drug efflux mediated by efflux transporters [4].

Efflux transporters in the gastrointestinal tract mainly include P-gp, MRPs and BCRP [5]. The distribution, expression quantities and spatial location of these efflux transporters are different in the human intestines. Intestinal efflux transporters are critical factors affecting oral absorption of drugs because these transporters can recognize, bind and excrete certain drugs into the intestinal lumen. Therefore, inhibiting the activity of intestinal membrane efflux transporters has gradually become a research hotspot to improve drug oral absorption [6,7]. It has been widely reported that many amphiphilic pharmaceutical excipients, such as D-a-tocopheryl polyethylene glycol 1000 succinate [6], Ployethylene glycols [8] and Pluronic [9], and small molecule compounds known as inhibitors positively suppress the activity of intestinal efflux transporters. The application of these excipients or inhibitors to prepare oral drug carriers can significantly improve oral drug absorption by reducing the intestinal drug efflux [10,11]. In addition, many novel nano-preparations including liposomes [12], microemulsions [13], solid lipid nanoparticles [7], and micelles [14], have significantly improved drugs oral absorption by inhibiting the activity of intestinal efflux transporters.

In this review, we systematically summarize intestinal efflux transporters and a variety of functional excipients and inhibitors that inhibit the activity of efflux transporters. Furthermore, plenty of cell and animal models for measuring drug efflux in vitro are summarized. Finally, different nanocarriers of improving drugs oral absorption are highlighted. In conclusion, we introduce various methods of inhibiting intestinal efflux transporters activity in detail, which has a significant reference for improving drugs oral absorption.

## 2. Intestinal Efflux Transporters

Small intestines of the human body are the main place for the absorption of nutrients and oral drugs. ATP-binding cassette (ABC) transporters are a class of membrane efflux transporters modulating the transport of drugs, exogenous and endogenous substances. Seven subfamilies (A-G) of ABC transporters have been found. Several different types of ABC membrane transporters distribute in intestinal epithelial cells, and these transporters could affect substances absorption by transporting substrates from enterocytes to intestinal lumen or blood in an ATP-dependent manner. Three efflux transporters of the ABC membrane proteins have been found on the lumen side of enterocytes, including P-gp [15], MRP2 [16] and BCRP [17], which function as an intestinal barrier due to them pumping drugs to intestinal lumen. MRP1-5 (other than MRP2) are on the basolateral side of enterocytes and actively transport oral substances from intestinal epithelial cells into the blood. Details of intestinal efflux transporters affecting substances absorption are described as follows.

### 2.1. P-gp

P-gp translated by ABCB1 gene (MDR1 gene) is one of ABC transporters in humans and rodents and widely distributes in the whole intestines [18]. The expression of P-gp in the intestines gradually increases from the duodenum to the colon and chiefly in the colon and distal small intestine [19]. Several nutrients and xenobiotics affect the expression of intestinal efflux transporters. The intestinal expression of P-gp obviously increased after rats received a fiber meal [20]. A wide variety of drugs such as paclitaxel, docetaxel, doxorubicin, and digoxin are not orally bioavailable due to intestinal P-gp pumps extruding these drugs from enterocytes to intestinal tract. To solve this problem, plenty of inhibitors (inhibiting P-gp activity or decreasing P-gp expression) such as verapamil, flavonoids, alkaloids, elacridar, tariquidar and zosuquidar, can be co-delivered with P-gp substrates to improve drug oral absorption by decreasing drugs efflux. More typical substances identified by P-gp or inhibiting P-gp activity are listed in Table 1.

### 2.2. MRPs

MRPs, the C subfamily of ABC transporters family, contain 9 proteins (MRP1-9), among which only MRP1-5 are related to the membrane transport of substances and have been reported to exist in intestine and colon of human [21]. Due to MRP2 special locality and relatively large expression quantities, it becomes one of the main intestinal efflux transporters that pumps certain substrates into the intestinal lumen. Intestinal MRP2 is to protect the organism from toxicants but it also affects some drugs absorption. It has been found that MRP2 is mainly expressed in the duodenum and jejunum, which may be related to its function [22]. Multiple factors modulate the expression of intestinal MRP2. Fructose-induced metabolic syndrome decreased intestinal MRP2 activity and expression, which could be reversed by geraniol and vitamin C [23]. The substrates of MRP2 include metabolites of endogenous and exogenous substances and organic anion compounds. More substrates and inhibitors of MRP2 are summarized in Table 1.

### 2.3. BCRP

BCRP is the second member of the G subfamily of ABC superfamily (ABCG2). Some substances can be pumped from enterocytes into the intestinal lumen by ABCG2. Therefore, ABCG2 can protect organisms from xenobiotics but it also reduces the oral absorption of drugs [24]. Studies have shown that BCRP extensively distributes in human intestines and its distribution is mainly concentrated in jejunal epithelial cells. Representative substrates and inhibitors of ABCG2 are summarized in Table 1. Researchers have found that some substrates (CYT387, Gefitinib, sorafenib etc.) of ABCG2 are also substrates of P-gp or MRP2 [24].

**Table 1:** Substrates and inhibitors of three main intestinal efflux transporters.

Transporters	Substrates	Inhibitors	Refs.
P-gp	digoxin, rhodamine-123, verapamil, rapamycin, cimetidine, silybin, atenolol, citalopram, mitoxantrone, doxorubicin, fexofenadine, rhodamine 123, aliskiren, betrixaban, celiprolol, paclitaxel and vincristine.	verapamil, cyclosporine A, elacridar, tariquidar, zosuquidar, alkaloids, flavonoids, pyrimidine aminobenzene derivatives, 4-indolyl quinazoline derivatives, quercetin, ivermectin, Royleanone, HM30181A, thilphenylbenzofuran derivatives, encequidar, CBT-1^®^.	[14,25,26,27,28,29,30,31,32,33,34,35,36]
MRP2	Valsartan, pravastatin, cisplatin, silybin, doxorubicin, sulfobromophthalein, dinitrophenyl-s-glutathione, calcein, methotrexate, ezetimibe glucuronide, resveratrol, etoposide, statins, and fexofenadine.	MK571, indomethacin, cyclosporin A, Nomegestrol acetate sulfated metabolites, indomethacin, ivermectin.	[31,37,38,39,40,41,42,43,44,45,46,47]
ABCG2	5-FU, silybin, zidovudine, cimetidine, nilotinib, bisantrene, ciprofloxacin, resveratrol, doxorubicin, mitoxantrone and topotecan.	pyrimidine aminobenzene derivatives, reserpine, Ko143, reserpine, ivermectin.	[38,41,43,44,45,48,49,50,51]

## 3. Models

### 3.1. Cell Models

In order to measure drugs efflux in vitro, several commonly used cell models such as Caco-2 monolayer and MDCK monolayer are well established [38]. The structure and function of Caco-2 monolayer and MDCK monolayer are consistent with the intestinal enterocytes. Since Caco-2 monolayer expresses a large number of efflux transporters, it is used to investigate whether certain small molecule compounds and excipients can inhibit efflux transporters activities [52,53]. In addition, MDCK monolayer and Caco-2 monolayer are also applied to investigate whether specific efflux transporters are involved in drugs transport [54].

#### 3.1.1. Caco-2 Monolayer

Caco-2 cells (20–80 generation) are derived from colon adenocarcinoma and differentiated into a complete polarized monolayer on day 21 and this monolayer is similar to the intestinal epithelial layer that contains brush borders, tight junctions and efflux transporters [55]. It has been confirmed that lots of efflux transporters including P-gp, MRP2 and BCRP distribute in Caco-2 cells. For Caco-2 monolayer, the expression of efflux transporters gradually increases along with culture time and reaches a stable level on day 21. When the transepithelial electrical resistance (TEER) of Caco-2 monolayer surpassed 200 Ω·cm^2^, it indicated that this monolayer could be used for studying the absorption and efflux of drugs [56].

**Figure 1:**
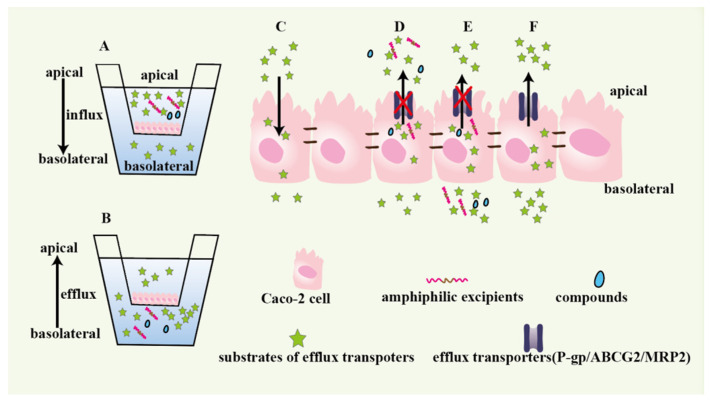
Caco-2 monolayers to evaluate the drug absorption and efflux and investigate functional excipients or inhibitors. A: the apical side is used as drugs donor to measure drugs absorption. B: the basolateral side used as drugs donor to research drugs efflux. C: drugs influx. D and E: studying whether compounds and excipients could prevent drugs from being pumped to the apical side of monolayer by efflux transporters. F: the drugs efflux mediated by efflux transporters.

The Caco-2 monolayer model composes of cells, apical side (A) and basolateral side (B). The transport direction of drug from B to A indicates drug efflux, while that of drug from A to B indicates drug influx. The apparent permeability coefficients P_app(A-B)_ and P_app(B-A)_ represent drugs absorption and efflux situation, respectively [52]. The drug efflux ratio is the value of P_app(B-A)_/P_app(A-B)_. When the value exceeds 2, it means that efflux transporters may participate in drugs transport [52]. The functions of Caco-2 monolayer are intuitively summarized in Figure 1, including measuring drug efflux and investigating whether functional excipients or compounds could inhibit efflux transporters activity [38,52,53,54]. Inhibitors of P-gp, MRP2 and BCRP were pretreated to Caco-2 monolayer, respectively, and then the efflux ratio of theaflavins obviously decreased compared to the control group (without adding inhibitors), which indicated that the three efflux transporters influenced theaflavins absorption [57]. Meropenem (MER) was loaded into nanoparticles packed Eudragit^®^ RSPO polymeric matrix to form nano-in-micro hierarchical microspheres (MER-RSPO) [58]. Compared to free MER solution, the MER efflux ratio for MER-RSPO significantly reduced from 2.62 to 0.35 in Caco-2 monolayer. In order to investigate whether poloxamines can inhibit P-gp pumps activity, Caco-2 monolayer pretreated with poloxamines was treated with doxorubicin (a P-gp substrate) [53]. The intracellular accumulated amounts of doxorubicin greatly increased, which indicated that poloxamines inhibited P-gp-mediated doxorubicin efflux. To explore more effective selective P-gp inhibitors, Caco-2 monolayer was pretreated with seven known P-gp inhibitors and then coped with Paclitaxel (PTX, a P-gp substrate) [52]. The results of PTX efflux ratio showed that LY335979 obviously selective reduced PTX efflux [52].

#### 3.1.2. MDCK Monolayer

The cultivation of Madin-Darby canine kidney (MDCK) monolayer is 2–4 days which is shorter than that of Caco-2 monolayer, and the TEER of MDCK monolayer is closer to that of human intestinal epithelium. The mechanism of measuring drug efflux MDCK monolayer is similar to Caco-2 monolayer. In addition, MDCK cells transfected with the gene of a specific efflux transporter could be used to research the effect of this transporter on drugs absorption and efflux [59]. MDCK cells are separately transfected with MDR1 gene, MRP2 gene and BCRP gene to obtain MDR1-MDCK, MRP2-MDCK and BCRP-MDCK cells that can express specific efflux transporters. MDCK monolayer can be used to explore substrates and inhibitors of efflux transporters. In Caco-2 monolayer and MRP2-MDCK/BCRP-MDCK monolayer, MRP2/BCPR inhibitors significantly decreased the efflux ratio of Silybin, which indicated that Silybin was the substrate of MRP2 and BCRP pumps [38]. Compared to free berberine hydrochloride (BBH, a P-gp substrate), the efflux ratio of BBH for the mixture of BBH and natural nanoparticles decreased from 25.8 to 17.1 in MDR1-MDCK monolayer, indicating that the nanoparticles could inhibit P-gp activity [60]. In addition, the application of MDCK monolayer to explore excipients of inhibiting efflux transporters activity is also advisable. BCRP-MDCK cells were used to research whether Tween 20 and Pluronic 85 could effectively inhibit BCRP-mediated mitoxantrone efflux [61].

### 3.2. Everted Gut Sac Model

In addition to cell models, the everted gut sac model is an important model to study whether efflux transporters participate in drugs transport due to lots of efflux transporters distributing in intestinal epithelial cells. To research drugs transport from serosal side to mucosal side (drug efflux), a small intestine about 10 cm (duodenum/jejunum/ileum) is everted to make serosal side face inward and one end of intestine is ligated, and then 1 mL Krebs-Ringer buffer containing drugs is added to the inner serosal side. Finally, everted gut sac containing drugs solution is incubated at 37 °C in a 20 mL Krebs-Ringer buffer. The everted gut sac method was used to research the efflux of BBR-loaded natural nanoparticles (Nnps-BBR) and free BBR, and results showed the efflux of BBR significantly decreased for Nnps-BBR due to Nnps inhibiting P-gp activity [60]. According to the everted gut sac method, the efflux ratio of Red Globe Grape, Raspbeery and Blackberry was accurately measured to be 1.55, 0.98 and 1.38, respectively [62]. This model is a cheap and practical method to accurately determine intestinal drugs efflux ratio.

## 4. Excipients to Inhibit Efflux Transporters Activity

### 4.1. TPGS

**Figure 2:**
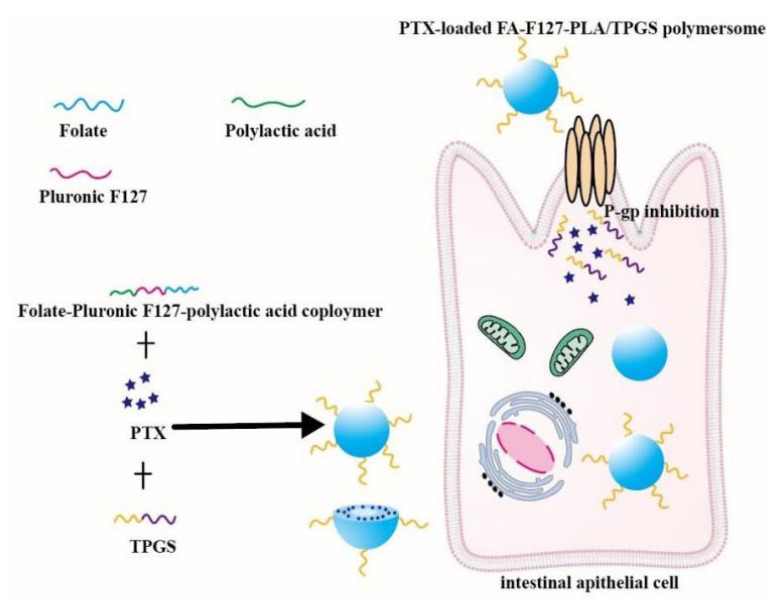
Schematic diagram of PTX-loaded FA-F127-PLA/TPGS mixed polymersome reversing the efflux mediated by P-gp. Remodified and summarized with permission from [70]. Copyright© 2019 Elsevier B.V.

D-a-tocopheryl polyethylene glycol 1000 succinate (TPGS) is a functional amphiphilic derivative of vitamin E and it can inhibit P-gp activity. In addition, TPGS is also an excellent agent that increases solubility of drugs and improves nano-formulations stability. The proven specific mechanisms of TPGS inhibiting P-gp include decreasing P-gp expressional amount, reducing mitochondrial membrane potential, depleting ATP and inhibiting P-gp-ATPase activity [63,64,65,66]. Owing to its P-gp inhibiting effect, amphiphilicity and degradability, TPGS has been widely used to prepare multiple nano-formulations to improve drug oral availability. Curcumin-loaded TPGS functionalized mesoporous nanocarriers outstandingly improved Curcumin oral bioavailability due to their small size and P-gp inhibition [6]. A nanocomplex composed of *N*-trimethyl chitosan and TPGS-modified poly (lactic-co-glycolic acid) was successfully prepared [67]. The oral bioavailability of the gemcitabine-loaded nanocomplex was 5.1 times higher than that of free gemcitabine due to P-gp inhibition. Microemulsion with TPGS as a surfactant was applied to deliver celecoxib, and the absorption of celecoxib greatly increased [68]. TPGS modified Daidzin-loaded zein nanoparticles were orally administrated to mice, and then the area under the curve (AUC) of the nanoparticles was wider and taller than that of Daidzin solution due to P-gp inhibition [69]. Oral PTX-loaded folate-conjugated Pluronic F127/polylactic acid polymersome modified by TPGS (PTX-loaded FA-F127-PLA/TPGS) were fabricated via a dialysis method. The AUC_0__–48h_ of PTX-loaded FA-F127-PLA/TPGS polymersome was 3737.14 ± 631.58 (ng/mL), while the AUC_0__–48h_ of Taxol^®^ was 559.18 ± 113.90 (ng/mL) [70]. The mechanism of PTX-loaded polymersome improving the PTX oral absorption was intuitively shown in Figure 2. In brief, TPGS is a promising oral nontoxic material and it could improve drug absorption by inhibiting P-gp activity and increasing solubility of insoluble drugs.

### 4.2. β-Cyclodextrin

β-Cyclodextrin (β-CD), a common polysaccharide, is usually used as a pharmaceutical ingredient and it has a hydrophilic outside and a lipophilic inside hole where hydrophobic drugs can be implanted [71,72]. In addition to improving drugs solubility, β-CD also inhibits P-gp by weakening P-gp-ATPase activity [73]. β-CD derivatives including Methyl-β-CD and Heptakis-β-CD have excellent inhibitory effect on P-gp activity [74]. In addition, β-CD also affects the activity of the drug metabolic enzyme CYP3A. Therefore, nanocarriers composed of β-CD and its derivatives have obtained more attention owning to them inhibiting P-gp activity and increasing drugs solubility. The Tacrolimus (KF506)-loaded hydroxypropyl-β-CD complexes were freeze-dried to form copolymer powders nanoparticles and its intestinal permeability value Papp significantly increased in inverted gut sac model, which was mainly due to these nanoparticles inhibiting P-gp activity [75]. Caco-2 cells were co-treated with R8-CM-β-CD (a derivatives of β-CD) and rhodamine-123 (a P-gp substrate), and then the internalized rhodamine-123 increased by 128%, which indicated that R8-CM-β-CD might have the ability of inhibit P-gp activity [76]. Insulin was loaded into R8-CM-β-CD to prepare a supramolecular complex (insulin/ R8-CM-β-CD) that showed excellent intestinal absorption, which might be attributed to that insulin/R8-CM-β-CD inhibited P-gp activity and improved intestinal insulin permeability [76]. The efflux ratio of nintedanib (a P-gp substrate)-loaded SEB-β-CD (a β-CD derivative) complex was 6–8 times lower than that of free nintedanib solution, which might be attributed to SEB-β-CD complex increasing nintedanib solubility and reducing P-gp activity [77]. Owning to it inhibiting P-gp activity and increasing solubility of drugs, β-CD is an ideal functional excipient improving drugs oral absorption.

### 4.3. Pluronic

**Figure 3:**
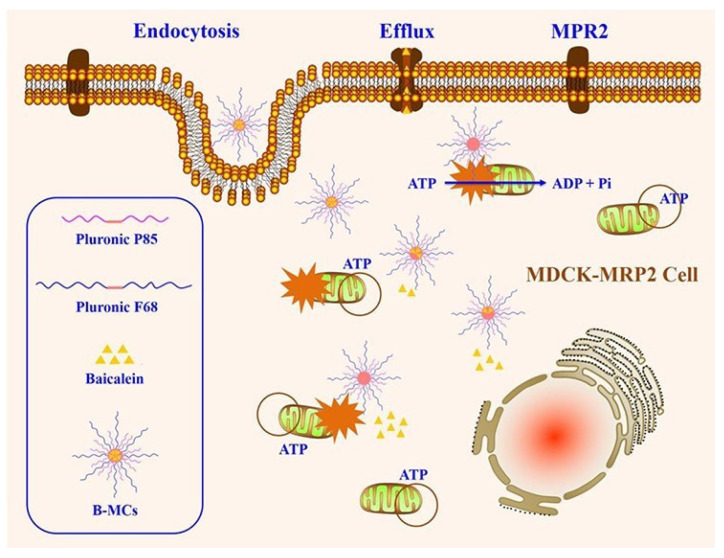
Schematic illustration of the mechanism on overcoming MRP2 of B-MCs. Reprinted with permission from [83]. Copyright© 2017 American Chemical Society.

Pluronic is an amphiphilic excipient that composes of a central hydrophobic chain with two hydrophilic chains attached by the side, and it usually function as stabilizer and surfactant. More importantly, it has been found that Pluronic copolymers could modulate efflux transporters activity [78]. P-gp activity can be inhibited by many types of Pluronic such as Pluronic 85, Pluronic F127, Pluronic F-68, Pluronic L92 and Pluronic L61 [4,79,80,81]. Pluronic F127-grafted-chitosan (Pl-g-CH), a polymeric derivative of Pluronic F127, obviously increased intracellular fluorescence intensity of rhodamine-123, which proved that Pluronic F127 inhibited P-gp activity while the specific mechanisms of how to inhibit P-gp were not further studied [82]. Compared to digoxin (a P-gp substrate) alone, the intracellular amounts of digoxin in LLC-PK1-P-gp cells treated with digoxin and Pluronic 85/tween 80 obviously increased, indicating that Pluronic 85/tween 80 inhibited P-gp-mediated digoxin efflux [79]. As an efflux transporters inhibitor and amphiphilic agent, Pluronic has been applied to prepare various nano-formulations to improve drugs oral absorption. Baicalein-loaded Pluronic P85/F68 micelles (B-MCs) were employed to reverse MRP2-mediated efflux of baicalein (a MRP2 substrate) [83]. B-CMs decreased the intracellular mitochondrial membrane charge and ATP level of MDCK-MRP2 cells, indicating that MRP2 was inhibited by B-CMs and the mechanisms of B-MCs improving baicalein oral absorption were shown in Figure 3. In conclusion, Pluronic is an excellent excipient for improving drugs oral absorption.

### 4.4. PEGs

The molecular weights of Ployethylene glycols (PEGs) change from 200 to 35,000. In addition to improving drugs solubility, PEGs (PEG-400/PEG-2000/PEG-20000) were also found to inhibit P-gp-mediated-rhodamine-123 efflux in a concentration depended on manner while the concrete mechanisms of how PEGs affected P-gp function were not researched [84]. In the existence of PEG-400, the permeable ability of ganciclovir (a P-gp substrate) was obviously increased, which indicated that PEG-400 suppressed P-gp activity [81]. After fresh rat intestinal mucosa was treated with PEG-grafted polyethyleneimine (PEI) thiolated by γ-thibutyrolatone (PEG-g-PEI) co-polymer and Rhodamin-123, the accumulative absorption of Rhodamine-123 greatly increased compared to Rhodamine-123 alone, indicating that PEG-g-PEI co-polymer might be a novel material inhibiting P-gp function [85]. In general, PEGs and its derivatives serving as functional materials inhibiting P-gp are important for improving drugs oral absorption.

### 4.5. Others

In addition to the mentioned excipients, Tween 20, Brij 58, Tween 80, sodium carboxymethyl cellulose and Cremophor EL, explicitly inhibited P-gp efflux function in MDCK-MDR1 cells in a concentration-depended manner [8]. It was also found that Tween 20, Brij 30 and Cremophor EL inhibited P-gp and ABCG2 activity [86]. Docusate sodium, sodium lauryl sulfate and sucrose monolaurate obviously blocked the ABCG2 efflux activity [87]. Polysorbate 20 had an excellent inhibiting effect on P-gp [88,89]. As efflux transporters inhibitors and solubilizer, these materials can be used to prepare liposomes, micelles, nanoparticles and self-microemulsions to improve oral availability of drugs. It is necessary to find more amphiphilic excipients with the function of inhibiting intestinal efflux transporters activity, which would help researchers to better overcome intestinal drugs efflux. The well proven mechanisms of different excipients improving drug oral bioavailability are clearly concluded in Table 2.

## 5. Strategies to Reverse Inhibit Intestinal Drug Efflux

The intestinal drug efflux is one of the important factors impacting oral drug absorption. It is emergent for scientists to find more strategies to reverse intestinal drug efflux mediated by intestinal efflux transporters. In order to solve this problem, some feasible methods for inhibiting intestinal efflux transporters activity have been reported, including silencing transporters and utilizing functional excipients and inhibitors, and these methods are introduced in detail as follows.

### 5.1. Silencing Transporters

Efflux transporters existing at the apical side of enterocytes inhibit oral drug absorption. To solve this problem, it is advisable to directly silence intestinal efflux transporters or other proteins that are involved in regulating the expression of intestinal efflux transporters. At present, small RNA or RNA-related-enzyme inhibitors are frequently used to reduce these transporters expression. Short hairpin RNA was combined with lentiviral vector to transfect Caco-2 cells, and then the ABCG2 mRNA and protein of transfected cells sharply decreased, indicating that ABCG2 was silenced [91]. Gene knockout by CRISPR/CAS9 system was also available to silence P-gp transporter [92]. The expression amount of sphingomyelin synthase (SMS) and membrane efflux transporters (P-gp/MRP2) of Caco-2 cells significantly decreased after Caco-2 cells was treated with the siRNA of SMS, which indicated that SMS participated in modulating the expression of P-gp and MRP2 [93]. The results showed that SMS might be a new target for silencing intestinal efflux transporters. Although silencing efflux transporters is very promising, it is only practicable at the cellular and animal level and very difficult to achieve the aim of silencing intestinal efflux transporters in clinical practice. More efficient methods silencing intestinal efflux transporters are still needed to be found.

### 5.2. Inhibit Efflux Transporters by Excipients

**Figure 4:**
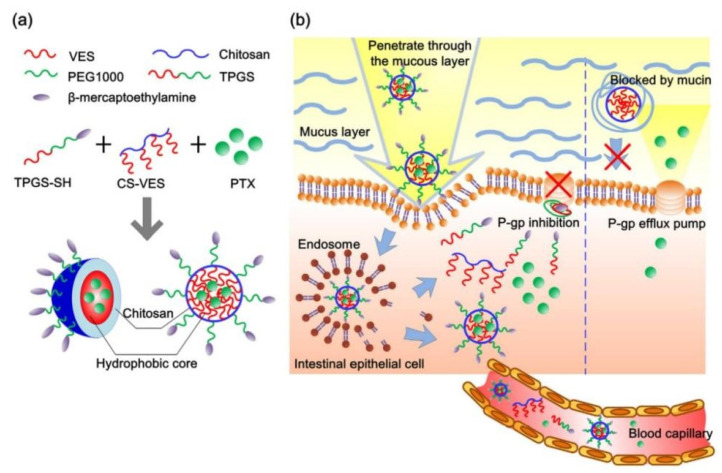
(**a**) Schematic drawing of self-assembled hybrid CS-VES/TPGS-SH nanomicelles in aqueous medium. (**b**) Schematic illustration of intestinal distribution of hybrid CS-VES/TPGS-SH nanomicelles, mucosal penetration, and subsequent P-gp inhibition for effective oral delivery of paclitaxel. Reprinted with permission from [94]. Copyright© 2017 Elsevier B.V.

A lot of amphipathic materials have been proved to inhibit P-gp activity including TPGS, β-CD, Pluronic, Tween 80, PEGs and Polysorbate 20. As P-gp inhibitors and stabilizers, these excipients have been widely used to prepare various nano-formulations to overcome intestinal drug efflux to improve oral drug absorption. Chitosan-vitamin E succinate copolymer (CS-VES) and β-mercaptoethylamine hydrochloride-modified succinylated TPGS (TPGS-SH) were synthesized to fabricate PTX-loaded CS-VES/TPGS-SH nanomicelles via a blank micellar drug delivery method [94]. In this research, the AUC_0–t_ of PTX-loaded CS-VES/TPGS-SH nanomicelles was 3.58 times higher than that of PTX-solution due to TPGS-SH inhibiting P-gp activity and chitosan increasing mucus adhesive ability, and these mechanisms of nanomicelles enhancing PTX oral absorption were shown in Figure 4. The nintedanib-loaded SEB-β-CD complex obviously decreased the efflux of nintedanib by inhibiting P-gp activity [77]. Compared to Taxol™, PTX nanocrystals stabilized by Pl-g-CH copolymer greatly improved PTX oral absorption due to Pl-g-CH inhibiting P-gp activity [82]. In conclusion, these amphiphilic materials can be used to fabricate micelles, nanoparticles and nanocrystals to overcome intestinal drug efflux.

### 5.3. Co-Delivery

In addition to silencing transporters and making use of excipients to inhibit the activity of intestinal efflux transporters, co-delivering inhibitors and drugs is also a practical method to inhibit the activity of intestinal efflux transporters and enhance oral drug absorption. Inhibitors of three main intestinal efflux membrane transporters are well summarized in Table 1 in detail. At present, three generation P-gp inhibitors have been found including Elacridar, Valspodar and Zosuquidar, while most of inhibitors could not inhibit P-gp activity during clinical trials [95]. More clinically efficient inhibitors are still needed to be found. Reasonable inhibitors of efflux transporters should meet the following requirements:

- effectively inhibiting the activity of several efflux transporters in cell and animal models;
- Being harmless for healthy cells;
- Having not influence metabolic enzymes activity;
- Efficiently inhibiting efflux transporters activities in clinical trials.

**Figure 5:**
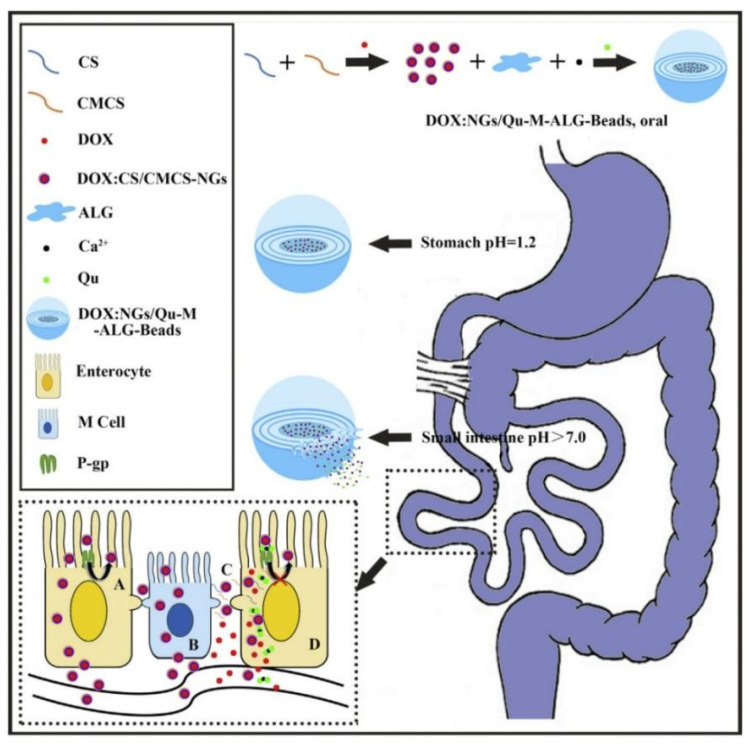
Schematic illustrating the effect of DOX: NGs/Qu-M-ALG-Beads on improving the oral absorption of DOX: A: transcellular pathway by pinocytosis, B: transcytosis by M cell, C: paracellular pathway by CS/CMCS-NGs induced tight junction opening and D: inhibition of P-gp in enterocyte by Qu. Reprinted with permission from [97]. Copyright© 2016 Elsevier B.V.

The absolute oral bioavailability of docetaxel (DTX) was 45.2% for a self-emulsifying drug delivery system that co-encapsulated DTX and Cyclosporine A (a P-gp inhibitor), 4.9% for DTX solution because this self-emulsifying drug delivery system reduced intestinal DTX efflux [96]. Chitosan (CS) and carboxymethyl chitosan (CMCS) was used to prepare doxorubicin (DOX)-loaded nanogels (DOX:CS/CMCS-NGs) which was further combined with quercetin (Qu) and sodium alginate (ALG) to fabricate multilayer DOX: NGs/Qu-M-ALG-Beads [97]. Results of in vitro drug release showed that DOX: NGs/Qu-M-ALG-Beads was stable in artificial gastric juice and it gradually released Qu in intestinal juice. In addition, the oral bioavailability of DOX: NGs/Qu-M-ALG-Beads was 18.56 times higher than that of free DOX due to nanogels being absorbed through the endocytic pathway and paracellular way and Qu inhibiting intestinal DOX efflux mediated by P-gp. The intestinal absorption mechanisms of DOX: NGs/Qu-M-ALG-Beads were shown in Figure 5.

## 6. Mechanism of Reversing Intestinal Drug Efflux

To reverse the intestinal drug efflux can be considered from improving dosage forms, increasing targeting ability and co-administrating drugs with inhibitors. Among these methods, improving dosage forms is a commonly used method to enhance drug oral bioavailability. Nanoparticles, micelles, liposomes and other nano-drug delivery forms are popular oral delivery systems for the following advantages: increasing drugs solubility, permeability and stability, and realizing the controlled release of drugs.

### 6.1. Liposomes

Liposomes are bilayer vesicles composed of phospholipid, cholesterol, antioxidant, surfactant and other basic materials, and its structure is similar to biofilm.

**Figure 6:**
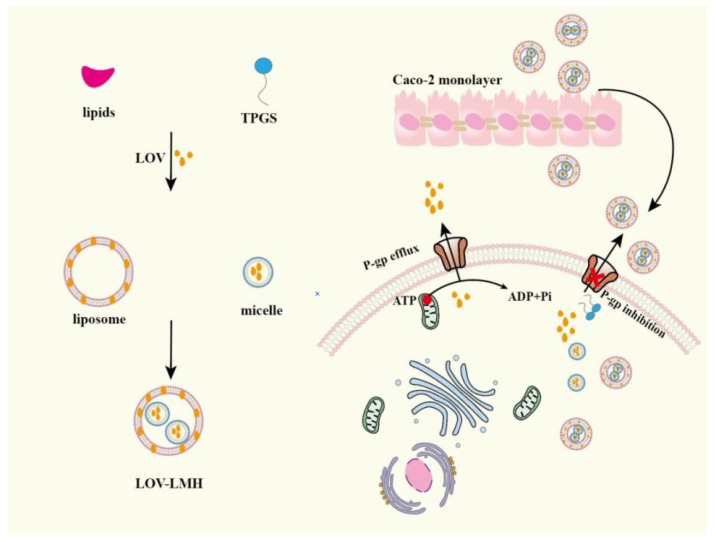
The mechanism of LOV-LMH improving the absorption of LOV in Caco-2 monolayer. Remodified and summarized with permission from [98]. Copyright© 2020 Elsevier B.V.

Drug-loaded liposomes are usually administrated by injection rather than by oral delivery method due to it being unstable in the gastrointestinal environment. Researching oral liposomes is necessary for its merits such as small particle size, improving insoluble drugs solubility and being easy to make. In order to achieve better oral absorption of drugs, many researchers have applicated functional excipients to prepare liposomes. These excipients endow liposomes with the ability of restraining P-gp activity, which makes liposomes become ideal drug oral carrier. Lovastatin (LOV)-loaded TPGS micelles were prepared via a solvent evaporation method and then these micelles were mixed with liposomes to fabricate LOV-loaded liposome-micelle-hybrid (LOV-LMH) [98]. LOV-LMH greatly improved the LOV permeability in Caco-2 monolayer by TPGS inhibiting P-gp-mediated LOV efflux, which was shown in Figure 6. LMH had great potential in oral delivering P-gp substrates. PTX-loaded-liposomes (composed of lipid, cholesterol and Pluronic F127) with its surface packaged by CS obviously improved PTX oral absorption by prolonging retention time and decreasing intestinal P-gp-mediated PTX efflux [99]. Positively charged glycol CS was used to package negatively charged Sorafenib (SF)-loaded liposomes to form GC-SF-Lip and then negatively charged Eudragit S100 was mixed with GC-SF-Lip to fabricate SGC-SF-lip [100]. SGC-SF-lip enhanced the permeability of drugs by the electrostatic interaction between positively charged GC-SF-lip and negatively charged gastrointestinal mucosa, and the in vivo pharmacokinetic experiments results showed that the AUC_0–t_ of SGC-SF-lip was 5.1 times higher than free SF group. Sohail, M.F et al. successfully fabricated DTX-loaded folate grafted thiolated CS nanoliposomes, the oral availability of these nanoliposomes was 13.6 folds compared to that of free DTX solution, which was attributed to thiomers inhibiting P-gp activity and thiolated CS prolonging intestinal retention time [101].

### 6.2. Solid Lipid Nanoparticles

Lipids are used as carriers to wrap drugs in lipid membranes to prepare Solid Lipid nanoparticles (SLNs) which accomplish the aim of controlling drug release, enhancing drugs solubility, increasing drug stability, improving drug loading efficiency and targeting at specific tissues and organs. In addition, SLNs were absorbed into intestinal lymphatic vessels by M cell phagocytosis, which avoided the liver first-pass effect [102].

**Figure 7:**
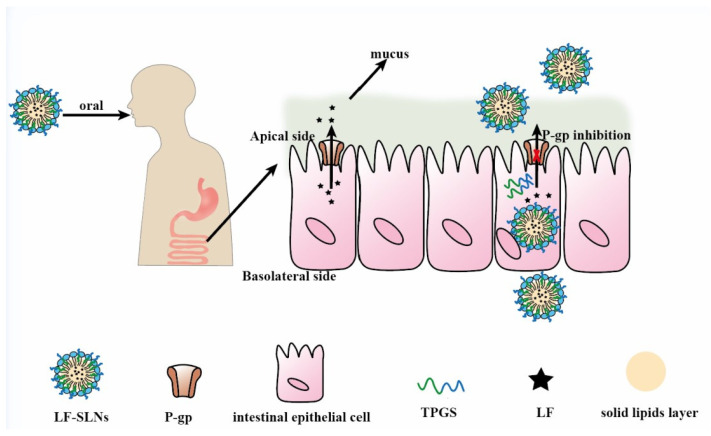
Mechanisms of LF-SLNs improving the oral bioavailability of LF. Remodified and summarized with permission from [103]. Copyright© 2016 Elsevier B.V.

To obtain ideal oral carriers, researchers have widely applied functional excipients to prepare novel SLNs, which endows these SLNs with P-gp inhibition. TPGS and Brij78 were used to fabricate curcumin (Cur)-loaded SLNs and results showed that the AUC_0–t_ of Cur-loaded SLNs was 12.27 times higher than that of free Cur [7]. Lumefantrine-loaded SLNs (LF-SLNs) with TPGS as stabilizer and P-gp inhibitor were prepared via a hot high shear homogenization ultrasonication method. The AUC_0__–t_ of LF-SLNs was 42,945.14 ± 2475.14 (h.ng/mL), while the AUC_0__–t_ of LF solution was 19,331.23 ± 1301.72 (h.ng/mL), which suggested that this SLNs could significantly improve LF bioavailability by inhibiting P-gp activity and the mechanism was shown in Figure 7 [103]. In addition to application of functional excipients, SLNs could improve drug oral absorption by increasing drug solubility and stability. Compared to the free camptothecin (CPT) group, the accumulative absorption of CPT-SS-PA-loaded SLNs in Caco-2 cells was significantly enhanced, which was attributed to the small size of SLNs and higher lipid solubility of CPT-SS-PA (a lipid derivative of CPT) [104]. Nunes’s research showed that SLNs obviously increased the stability of phenolic drugs, thus improving their oral bioavailability [105]. Although SLNs show great potential in oral drug delivery, they are unstable in gastrointestinal environment due to lipases degrading them. A lipase inhibitor orlistat (OLST) was incorporated into SLNs to load fluorescence (P2- or P4-OLST-SLNs), and the fluorescence intensity of P2-OLST-SLNs in blood was higher than SLNs without incorporation of OLST [106]. Lymphatic transport of P4 was 7.56% for OLST-SLNs and 1.27% for SLNs, which was attributed to OLST protecting OLST-SLNs from lipolysis thus resulting in more particles survival.

### 6.3. Nanoemulsion and Self-Emulsified Drug Delivery Systems

Nanoemulsion (NE) and self-emulsified drug delivery systems (SMEDDS) composed of two incompatible phases, surfactant and co-surfactant, are stable oil-water mixed systems with a particle size of 10–100 nm, both of which are ideal oral carriers improving drugs solubility and stability. To reverse intestinal drug efflux, besides their own merits, the application of functional excipients or inhibitors to prepare NE and SMEDDS is a feasible method.

**Figure 8:**
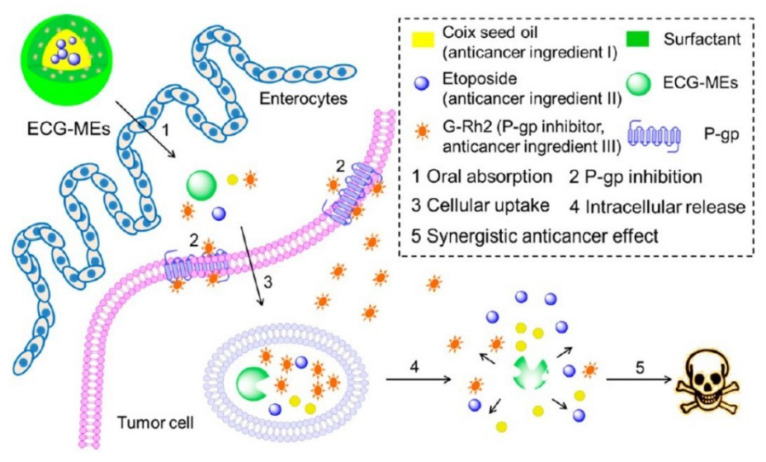
Schematic design of multicomponent microemulsions, ECG-MEs, for oral delivery of anticancer drug to overcome the MDR tumor. Reprinted with permission from [13]. Copyright© American Chemical Society.

Honokiol (a P-gp inhibitor) was encapsulated into SMEDDS with sirolimus, and the efflux ratio of sirolimus was about 35-folds lower than sirolimus-loaded SMEDDS without incorporation of honokiol [107]. NE could improve drugs oral absorption by improving solubility and permeability of drugs and intestinal lymphatic transport. Compared to BBH suspension, the oral relative bioavailability of BBH-loaded NE was 440.40%, which was attributed to NE improving the intestinal permeability and solubility of BBH [108]. The oral C_max_ of methotrexate was 81.72 ± 23.01 (ng/mL) for methotrexate-loaded NE, 12.13 ± 3.38 (ng/mL) for free methotrexate, which was due to methotrexate-loaded NE being absorbed into lymphatic vessel [109]. NE-loaded CS sponges with two merits including improving intestinal mucoadhesive properties and drugs permeability might have potential for orally delivering drugs [110]. In addition to making use of its own merits, application of inhibitors to prepare NE is an advisable method for improving drugs absorption. In addition to possessing anticancer effect, Ginsenoside Rh2 (G-Rh2) and coix seed oil were also separately used as mimic surfactant and oil phase to fabricate etoposide-loaded microemulsions (ECG-MEs) [13]. ECG-MEs obviously improved etoposide oral absorption and the in vivo anticancer results showed the tumor growth inhibition rate was 70.7% for ECG-MEs, 36.1% for EC-MEs without G-Rh2. To research the possible mechanisms of ECG-MEs improving drug oral absorption, the P-gp expression and its ATPase activity were studied. The ΔRLU (relative light unit) value obviously increased after MCF-7/MDR cells treated with G-Rh2 or ECG-MEs, which indicated that G-Rh2 inhibited P-gp activity by activating its ATPase activity. In addition, the in vivo imaging results showed that ECG-MEs could efficiently accumulate at the tumor site by the EPR effect. These mechanisms about ECG-MEs facilitating etoposide oral absorption were shown in Figure 8.

### 6.4. Polymer Micelles

Synthetic amphiphilic copolymers spontaneously self-assemble in water to form polymer micelle with a size of 1–100 nm to encapsulate drugs, which would greatly improve drugs solubility, stability and oral bioavailability [111,112,113].

**Figure 9:**
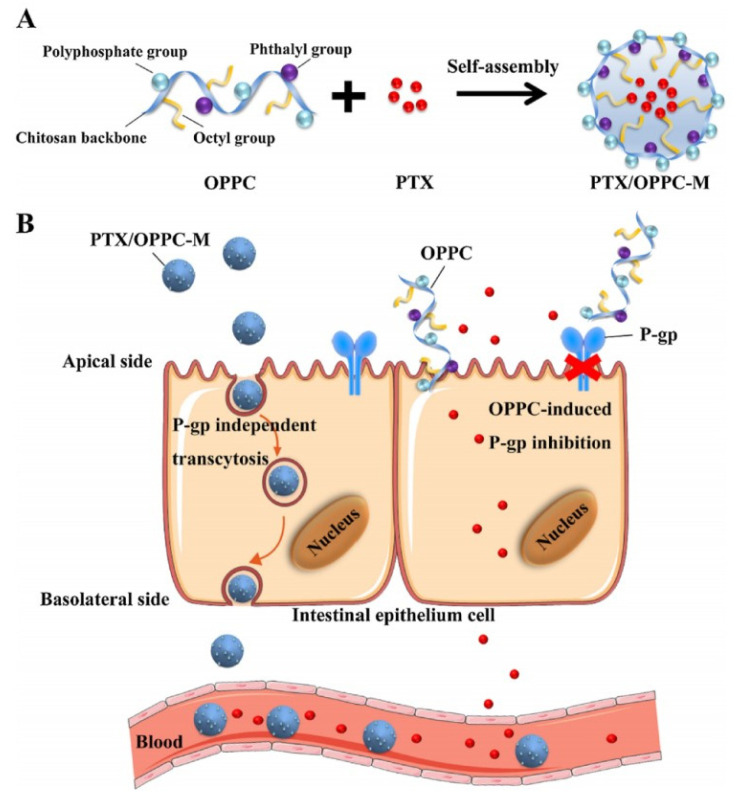
Scheme of the effect of OPPC micelles on improving the oral absorption of PTX. (**A**) The structure of OPPC and the strategy of constructing the PTX/OPPC micelles; (**B**) The hypothetical mechanism of improved oral absorption of PTX by OPPC micelles. Reprinted with permission from [14]. Copyright© 2018 Elsevier Ltd.

To increase oral absorption by reversing intestinal drug efflux, taking functional excipients or inhibitors to prepare drug-loaded micelles is a feasible method. Berberine-loaded micelles composed of Pluronic 85 and Tween 80 [79]. The AUC_0__–t_ of Thalifendine-Glu, a metabolite of berberine in plasma, was 20,371 ± 12,753 (ng.min/mL) for berberine-loaded micelles, 4269 ± 1680 (ng.min/mL) for free berberine, which suggested that the micelles obviously improved the oral availability of berberine due to Pluronic 85 and Tween 80 inhibiting P-gp activity [79]. A new CS derivative (OPPC) was used to fabricate micelles to encapsulate PTX (PTX/OPPC) and the AUC_0–24h_ of PTX/OPPC was 6.75 ± 1.66 (ug.h/mL), while that of Taxol^®^ was 1.22 ± 0.48 (ug.h/mL), which was attributed to OPPC decreasing P-gp activity [14]. The concrete mechanisms of PTX/OPPC micelles improving drug oral absorption included transcytosis and P-gp inhibition, which were shown in Figure 9. To improve drugs absorption, it is also advisable to endow micelles with the ability of targeting and binding intestinal absorptive transporters. The oral absorption of Valine/phenylalanine grafted-Cur-loaded TPGS derivatives polymeric micelles was 10.50-fold greater than that of Cur solution, which was mainly attributed to that these micelles actively combined with Peptide transporter 1 (PepT1, an absorptive transporter protein of intestinal epithelium) and inhibited P-gp-activity to improve drug absorption [114]. Qu-loaded CS micelles might be ideal oral carriers for it improving drugs oral absorption by inhibiting P-gp and opening the tight junction [10]. Thioglycolic acid-modified CS (SH-OCG) was synthesized to prepare PTX-loaded SH-OGC micelles that had a great potential in oral drug delivery due to it inhibiting P-gp efflux, unfolding intestinal tight junctions and increasing intestinal mucosa adhesive capacity [115].

### 6.5. Nanoparticles

Nanoparticles (NPs) have so many advantages such as improving drugs solubility, increasing drugs stability and small size that NPs become a research hot area for orally delivering drugs. Utilizing functional excipients to endow NPs with the properties of mucous-adhesive ability or P-gp inhibition is an advisable method to improve drugs oral absorption. Wang, Q. et al. added poly (vinyl methyl ether/maleic anhydride) (PVMMA) to prepare PTX-loaded NPs (PTX-m-NPs) via the emulsification solvent evaporation method [116]. The AUC_0–t_ of PTX was 62.2 ± 10.4 (ng.h/mL) for PTX-m-NPs, 30.8 ± 5.5 (ng.h/mL) for PTX-loaded NPs without PVMMA, 29.3 ± 7.8 (ng.h/mL) for Taxol^®^, which indicated that NPs composed of bioadhesive PVMMA excipient had great ability to improve oral bioavailability of drugs by prolonging intestinal retention time. It was found that Brij-grafted-CS (BC12) significantly decreased the intracellular ATP level of MDCK-MDR1 cells [117]. The TEER of MDCK-MDR1 monolayer treated with Berberine-loaded-BC12 NPs significantly decreased, which indicated that BC12 NPs opened the tight junctions. In conclusion, BC12 NPs might become ideal oral carriers that improve insoluble P-gp substrates absorption by inhibiting P-gp and opening tight junctions. The bioavailability of topotecan-loaded core-shell lipid NPs was 1.62-folds compared to free topotecan due to intestinal lymphatic transport of lipid NPs [118].

### 6.6. Nanocrystals

Nanocrystals (NCs) are stable dispersion system formed by drug particles and a small amount of surfactant or polymer materials. NCs gradually become ideal oral carriers due to its high drug encapsulation rate and loading ratio, P-gp inhibition and prolonged retention time.

**Figure 10:**
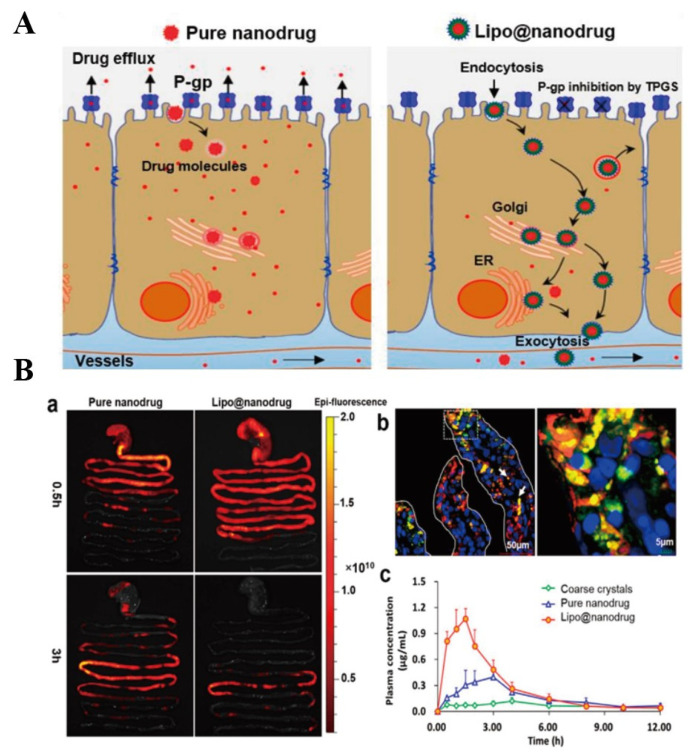
(**A**): Schematic illustration of potential transport mechanisms of pure nanodrug and Lipo@nanodrug. (**B**): In vivo absorption of SQV formulations. (**a**) Ex vivo imaging of NP transport through the rat GI tract at different time points; (**b**) fluorescence staining examination of intestinal mucosa absorption of Lipo@nanodrug 2 h after oral administration; and (**c**) mean SQV concentration in plasma over time after oral administration of coarse crystals, pure nanodrug, and Lipo@nanodrug (*n* = 5). Reprinted with permission from [119]. Copyright© 2017 Elsevier B.V.

Pluronic-CS co-polymer was synthesized to prepare PTX-loaded NCs [82]. In comparison to Taxol™, PTX-loaded NCs showed better oral absorption and anti-tumor effect because NCs improved the solubility of PTX, prolonged retention time through the electrostatic interaction between CS and intestinal mucosa, opened tight junctions and inhibited P-gp efflux [82]. NCs are degraded in gastrointestinal environment. Thus, protecting NCs from being degraded is an advisable method to improve drug-loaded NCs absorption. A phospholipid bilayer (containing TPGS) was used to encapsulate saquinavir (SQV, a P-gp substrate) pure drug NPs to form Lipo@nanodrug [119]. It was found that Lipo@nanodrug mainly located at the endoplasmic reticulum and Golgi apparatus of Caco-2 cells in intact nanostructure. Results of in vivo imaging showed that the absorption of Lipo@nanodrug was faster than pure drug NPs after 3 h of SD rats received these nanoparticles. In addition, the oral bioavailability of Lipo@nanodrug was 1.77-fold higher than pure drug NPs, which was due to that Lipo@nanodrug inhibited intestinal P-gp activity, prevented the drug releasing form pure drug NPs and improved lipid raft mediated transcytosis, and these mechanisms and results were clearly shown in Figure 10. More efficient oral NCs are still needed to be fabricated for improving drugs oral availability.

## 7. Conclusions

Oral administration is an ideal option for patients to accept treatment, by which patients would bear less financial burden and have better compliance. However, the oral bioavailability of some drugs is limited due to intestinal efflux transporters-mediated drug efflux. To overcome the poor oral absorption of drugs caused by intestinal efflux transporters, we comprehensively summarize typical intestinal efflux transporters including P-gp, MRP2 and ABCG2. In addition, lots of amphiphilic materials such as Pluronic 85, Tween 20 and TPGS that inhibit efflux transporters activity have been introduced in detail. Furthermore, plenty of intestinal efflux transporters inhibitors have been introduced including cyclosporine A, HM30181A and pyrimidine. Finally, we systematically summarize various nano-formulations that successfully improve drugs oral absorption by decreasing the activity of intestinal efflux transporters. Our work would help researchers to better understand the effect of intestinal efflux transporters on drugs oral absorption. In conclusion, this review has a significant reference for reversing intestinal drug efflux to improve oral drug absorption.

## Figures and Tables

**Table 2:** Excipients to enhance oral drug bioavailability.

Materials	Mechanism of Improving Oral Drug Bioavailability	Refs.
TPGS	Inhibiting P-gp and increasing solubility of insoluble drugs.	[63,64,65,66]
PEGs	Inhibiting drugs efflux mediated by P-gp.	[81]
β-CD	Reducing P-gp activity and improving solubility of insoluble drugs.	[71,72,74]
Pluronic	Inhibiting the activity of MRP2 and P-gp.	[82,83,90]
Polysorbate 20	Inhibiting P-gp activity.	[88,89]
Tween 20	Inhibiting drugs efflux mediated ABCG2 and P-gp.	[86]
Tween 80	Inhibiting P-gp activity.	[8]
Docusate sodium, sodium lauryl sulfate and sucrose monolaurate	Increasing the absorption of ABCG2 substrates.	[87]
Cremophor EL	Inhibiting ABCG2 and P-gp activities.	[86]
Brij 30/58	Reducing activities of P-gp and ABCG2.	[8,86]
